# Influence of Restrictions During COVID-19 Pandemic on Physical Activity and Quality of Life in Cardiovascular, Kidney Transplant and Healthy Adults

**DOI:** 10.3390/ijerph23030323

**Published:** 2026-03-05

**Authors:** Lena Grams, Momme Kueck, Thorben Sundermeier, Sven Haufe, Anne-Katrin Nelius, Arno Kerling, Uwe Tegtbur, Alexander A. Albrecht

**Affiliations:** Clinic for Rehabilitation and Sports Medicine, Hannover Medical School, Carl-Neuberg-Str. 1, 30625 Hannover, Lower Saxony, Germany; kueck.momme@mh-hannover.de (M.K.); sundermeier.thorben@mh-hannover.de (T.S.); haufe.sven@mh-hannover.de (S.H.); nelius.anne-katrin@mh-hannover.de (A.-K.N.); tegtbur.uwe@mh-hannover.de (U.T.);

**Keywords:** COVID-19, heart disease, KTx, mental health, physical activity guidelines, SF-36

## Abstract

**Highlights:**

**Public health relevance—How does this work relate to a public health issue?**

- This study Investigates the effects of pandemic-related restrictions on physical activity and quality of life, key determinants of public health.
- It provides evidence on particularly vulnerable groups (rehabilitation patients) during social crises.

**Public health significance—Why is this work of significance to public health?**

- It shows that sufficient physical activity (>150 min/week) can often be maintained even under lockdown conditions.
- It identifies rehabilitation patients as a high-risk group for greater loss of activity and thus potential consequential risks.

**Public health implications—What are the key implications or messages for practitioners, policy makers and/or researchers in public health?**

- Rehabilitation and aftercare programs should integrate targeted adaptive exercise options (e.g., digital or local formats) in times of crisis.
- Health policy measures should be tailored to vulnerable patient groups in order to minimize declines in activity and long-term health consequences.

**Abstract:**

Background: During the COVID-19 restrictions in Germany, individuals were permitted to engage in individual physical activity (PA). However, limited information exists regarding the impact of these restrictions on PA and mental health. The aims of this study were to identify changes in PA behavior and Quality of Life (QoL) during the Corona-Virus restrictions to develop strategies to mitigate negative health effects. Methods: Internet-based questionnaires on PA and QoL were distributed to outpatients with heart disease (Reha), participants in an in-clinic rehabilitation program, patients enrolled in a structured post-kidney transplant program (KTx), and healthy adults (HG). Results: Compared to pre-lockdown levels, all groups experienced a significant reduction in daily and sporting physical activity (all *p* < 0.05). Reha participants showed a significantly greater reduction in sporting activity compared to KTx and HG (*p* < 0.05). However, 88.3% (Reha), 85.5% (KTx), and 92.6% (HG) met the WHO recommendation of ≥150 min/week of moderate activity (*p* = 0.001). No significant differences were observed in the mental sum score of the SF-36 between groups (*p* = 0.263). Conclusions: The majority of individuals managed to maintain sufficient PA levels even during the lockdown. However, Reha participants appeared to experience the greatest burdens, leading to a more pronounced reduction in sports activity.

## 1. Introduction

Coronavirus disease (COVID-19), initially identified in China in December 2019, is a highly transmissible illness caused by the severe acute respiratory syndrome coronavirus 2 (SARS-CoV-2) [1]. The impact of SARS-CoV-2 on human health extends beyond respiratory complications, encompassing immune system suppression, exacerbation of underlying medical conditions, and potential systemic failure leading to mortality [2]. As of June 2024, the pandemic has resulted in 775,645,882 confirmed cases and 7,102,636 million deaths worldwide [3].

In early 2020, governments globally implemented stringent measures to curb the spread of the virus, including social distancing protocols aimed at minimizing close human contact whenever feasible. In some nations like Spain and Italy, individuals were mandated to observe strict stay-at-home orders, further impeding participation in physical activities. Consequently, hospitalizations surged worldwide, and billions of individuals were confined to their homes, inadvertently fostering sedentary behaviors and precipitating significant adverse health effects among both infected individuals and the general populace [4,5,6]. Given that physical inactivity ranks as the fourth leading risk factor for global mortality, responsible for approximately 3.2 million deaths annually [7], addressing this issue is imperative for patients and healthy adults alike.

With gyms, sports clubs, and outpatient rehabilitation facilities shuttered in Germany for approximately three months, it is crucial to ascertain how patients and healthy adults compensated for reduced daily physical activity during COVID-19 restrictions to meet the World Health Organization’s (WHO) recommendations of at least 150 to 300 min of moderate-intensity physical activity per week [8]. Reduced physical activity [9,10] and mental health challenges, including perceived stress and maladaptive coping mechanisms due to life uncertainties (e.g., economic or social uncertainties), have been documented during lockdown periods [11,12]. However, it remains unclear whether outpatients previously engaged in structured in-clinic rehabilitation programs or home-based training interventions employ different strategies to maintain physical activity levels and mental well-being.

Hence, the objectives of this study were to discern changes in physical activity behavior and mental health status during COVID-19 restrictions in Germany across three distinct groups, with the aim of formulating strategies to mitigate long-term adverse health outcomes.

## 2. Materials and Methods

After obtaining institutional review board approval, this study was conducted in adherence to the principles outlined in the Helsinki Declaration and the European Union’s Convention on Human Rights and Biomedicine. Approval for the study was obtained from the Ethics Committee of the Hannover Medical School, and written informed consent was obtained from all participants prior to enrollment.

**Figure 1:**
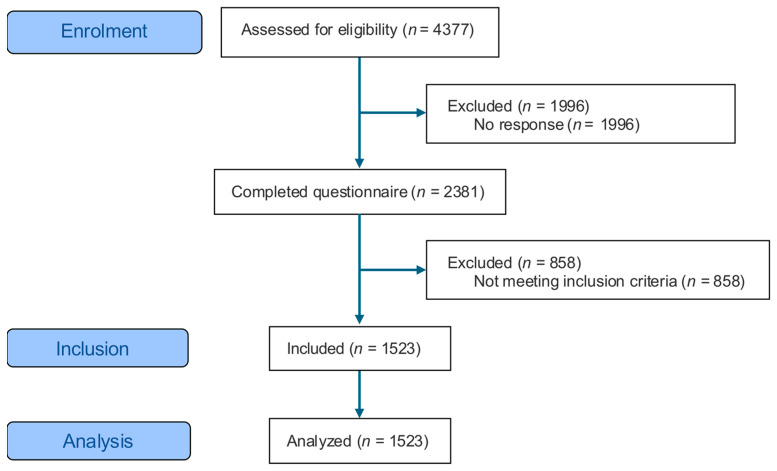
Participants.

The survey was administered during the COVID-19 lockdown period in May and June 2020. The participants were recruited from the following sources: studies of the Clinic of Rehabilitation and Sports Medicine, outpatients of the Clinic of Rehabilitation and Sports Medicine who were participating directly before the lockdown period in an in-clinic rehabilitation program, and students and employees of the Hannover Medical School. Participants were requested to complete an internet-based questionnaire. In cases where online participation was not feasible, the questionnaire was distributed via postal mail. The recruitment process involved three distinct participant groups. The first group, referred to as the rehabilitation group (Reha), comprised outpatients diagnosed with heart disease who had been engaged in an in-clinic rehabilitation program involving endurance and weight training once or twice a week prior to the lockdown. The second group, designated as the patient group (KTx), consisted of individuals enrolled in the structured post-kidney transplant program known as KTx360° [12,13] which continued during the lockdown period. The third group, denoted as the healthy group (HG), comprised individuals who reported no history of disease based on responses to questionnaire items. The inclusion criterion was adulthood, defined as being at least 18 years old. Exclusion criteria encompassed active COVID-19 infection or any other medical condition apart from documented heart disease or participation in the KTx program that could potentially impact physical activity during the lockdown. Of the 2381 questionnaires that were completed, 1523 were suitable for analysis (see Figure 1).

The questionnaire comprised three components: the Freiburg Questionnaire (FrQ) for assessing physical activity, the Short-form 36 (SF-36) questionnaire for evaluating quality of life (QoL), and self-designed COVID-19 specific inquiries. The FrQ, developed and validated by Frey and colleagues [14], is employed to assess both everyday activities—categorized into daily and leisure activities—and sporting activities over the course of a week. It comprises eight sets of questions, each focused on type, duration, and intensity of activity, and reports physical activity levels in metabolic equivalents of task (MET)-hours per week [15]. Based on the scores obtained, participants were categorized as “far too little active,” “minimum requirement fulfilled,” or “sufficiently active.” Additionally, the time spent engaged in moderate activity (≥3 MET and <6 MET) and vigorous activity (≥3 MET) per week was calculated for comparison with the WHO guideline of at least 150 min of moderate activity per week [15].

The SF-36 questionnaire, designed to measure health-related quality of life (QoL) [16], comprises 36 questions across eight dimensions, yielding both physical and mental sum scores. The eight dimensions are as follows:

1. General health perception,
2. Physical health,
3. Restricted physical role function,
4. Physical pain,
5. Vitality,
6. Mental health,
7. Restricted emotional role function,
8. Social functioning.

The COVID-19-specific questionnaire encompassed self-constructed inquiries regarding perceived restrictions and burdens attributed to the COVID-19 pandemic. Due to the novelty of the SARS-CoV-19 virus, no validated questionnaire addressing pandemic-related influences was available. The questionnaire solicited personal data, inquiries about health status, and details regarding physical activity and transportation patterns before and during the lockdown. Daily physical activity and sporting activity were rated on a scale of 0–10 (ranging from no activity to very high activity), with participants also asked to provide reasons and perceived burdens associated with changes in activity during the lockdown (see Supplementary File S1).

Due to the nature and timeline of the included studies, a proportion of participants in the KTx and HG groups had completed a FrQ or SF-36 assessment within one year prior to the survey. Consequently, a pre–post analysis was conducted on the results obtained from both the pre-lockdown and lockdown periods. This study was conducted and reported in accordance with the STROBE (Strengthening the Reporting of Observational Studies in Epidemiology) guidelines for cross-sectional studies.

### Statistical Analyses

All data are presented as mean ± standard deviation. Normal distribution was assessed using the Kolmogorov–Smirnov test. The distribution of the data was further examined using a chi-squared test with Cramér’s V as effect size. In this context, 0.1 represents a small effect, 0.3 a medium effect, and 0.5 a large effect. Differences between two time points were evaluated using a paired t-test for parametric data or a Wilcoxon test for non-parametric data. A linear correlation between activity level (FrQ sum of activities) and QoL scores was conducted using Pearson’s correlation for parametric data and Spearman’s for non-parametric data. Group differences across all groups were analyzed using ANOVA. The interaction of time and group in the pre–post analysis was assessed using ANOVA with repeated measurements. In both cases, the eta-squared η^2^ was determined to be the effect size, with 0.01 representing a small effect, 0.06 a medium effect, and 0.14 a large effect. All post hoc tests were adjusted according to Bonferroni. The significance level was set at 0.05. All statistical analyses were conducted using SPSS (version 27, Armonk, NY, USA).

## 3. Results

A total of 1523 participants were included in the analysis, distributed across three groups. The groups exhibited significant differences in age, gender, occupation, and residential area (see Table 1). The Reha group was characterized as the oldest cohort with the highest proportion reporting no occupation (see Table 1). The HG group represented the youngest cohort with the highest percentage engaged in sedentary occupations, while the KTx group had the highest proportion residing in rural areas (see Table 1).

Subjectively assessed daily physical activity was significantly higher in the HG group (*p* < 0.001) compared to both the Reha and KTx groups (post hoc *p* < 0.001 for both). There were significant differences in physical activity between all groups (*p* < 0.001), with the KTx group demonstrating the lowest score (post hoc *p* < 0.001 vs. Reha, *p* < 0.001 vs. HG) and the HG group exhibiting the highest score (post hoc *p* = 0.023 vs. Reha).

**Figure 2:**
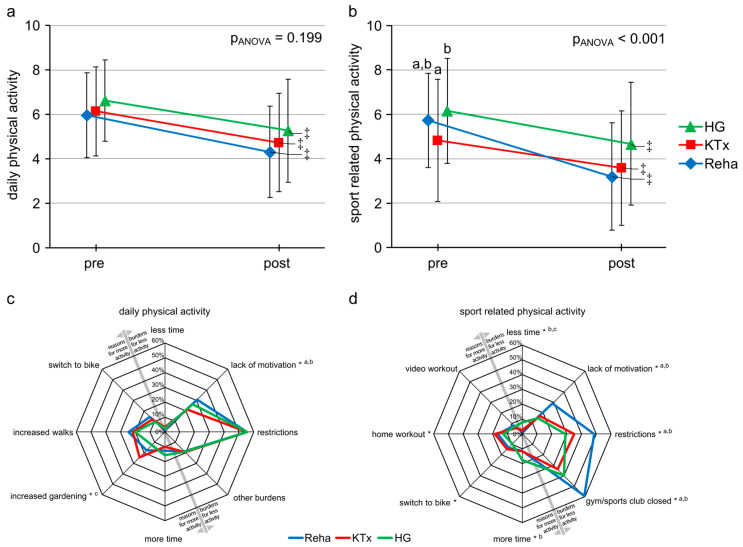
(**a**–**d**): Daily physical activity (**a**) and sport-related physical activity (**b**) assessed on a scale from 0–10; reasons and burdens for increased daily physical activity (**c**) and sport-related physical activity (**d**) during lockdown, presented as percentages. Pre: before lockdown; post: at the end of lockdown; ‡ *p* < 0.05 pre vs. post within each group; * *p* < 0.05 between the three groups; post hoc tests: ^a^ Reha vs. KTx *p* < 0.05, ^b^ Reha vs. HG *p* < 0.05.

Compared to pre-lockdown levels, activity at the end of the lockdown period showed a significant reduction in all groups for both daily and sporting activities (see Figure 2). There were no significant differences between groups for daily physical activity. However, the Reha group exhibited a greater reduction in sportive activity compared to both the KTx and HG groups (see Figure 2a,b).

For all groups, the primary burden leading to reduced daily physical activity was the lockdown restrictions, followed by lack of motivation, with Reha exhibiting significantly lower motivation levels than KTx and HG (see Figure 2c). Increased gardening, walking, and cycling were common reasons for augmented daily activity across all groups, with no significant differences except for significantly more gardening among KTx compared to Reha and HG (see Figure 2c). Concerning sporting activity, lack of motivation, restrictions, and closure of gyms/sports clubs were the predominant barriers, with Reha showing significantly higher values than KTx and HG (see Figure 2d). Reasons cited for increased sporting activity, such as having more time, switching to cycling, and engaging in home workouts, exhibited significant differences across all groups, though post hoc tests identified only a significant difference in increased time between Reha and HG (see Figure 2d).

The results of the FrQ revealed significant differences between the groups in total activity scores and classifications (see Table 2). HG demonstrated significantly higher total scores than Reha and KTx, as well as greater engagement in sporting activities (see Table 2). Leisure activity was notably lower among KTx participants (see Table 2).

Regarding adherence to the WHO guidelines for physical activity, 88.3% of Reha participants, 85.5% of KTx participants, and 92.6% of HG participants (post hoc: Reha vs. HG *p* = 0.020; KTx vs. HG *p* < 0.001) achieved more than 150 min per week of moderate intensity activity (*p* = 0.001, Cramér’s V = 0.10). There were no significant differences between the groups in the time spent in moderate activity or moderate sporting activity (see Table 3). However, HG participants spent more time engaging in vigorous sporting activities compared to Reha and KTx participants (see Table 3).

Quality of Life (QoL) exhibited significant variations between groups in terms of the physical sum score, with HG participants displaying a higher score compared to both Reha and KTx participants (see Table 4). Correlation analyses between activity levels (FrQ sum of activities) and QoL revealed significant correlations for the physical sum score in the Reha group (r = 0.22, *p* < 0.001) and the KTx group (r = 0.19, *p* = 0.004), as well as for the mental sum score in the HG group (r = 0.12, *p* < 0.001).

The pre–post analysis conducted on the KTx and HG groups, within one year prior to the survey and during the lockdown period, revealed a significant increase in physical activity in terms of both the sum of activities and everyday activities for the KTx group compared to the HG group (see Table 5). However, there were no significant differences observed over time in terms of Quality of Life (QoL) between the KTx and HG groups (see Table 5).

## 4. Discussion

### 4.1. Summary of Key Findings

This study examined changes in physical activity (PA) and quality of life (QoL) during the first COVID-19 lockdown in Germany among cardiac rehabilitation patients (Reha), kidney transplant patients enrolled in a structured follow-up program (KTx), and healthy adults (HG). Both daily and sporting activity declined significantly across all groups. The most pronounced group differences were observed for sport-related physical activity, with Reha participants showing a greater reduction compared to KTx and HG. Despite these reductions, the majority of participants achieved the World Health Organization (WHO) recommendation of ≥150 min of moderate activity per week [8], although healthy adults were significantly more likely to meet this threshold than patient groups. QoL remained largely stable, with differences observed only in the physical sum score and no significant pre–post changes detected in the subgroup analysis.

### 4.2. Interpretation of the Reduction in Physical Activity

The observed reduction in PA during lockdown aligns with international reports documenting decreased physical activity and increased sedentary behavior during COVID-19 restrictions [9,10,18,19,20,21]. Particularly among individuals with chronic diseases, lockdown measures have been associated with significant declines in habitual activity [22,23].

In the present study, both daily and sporting activity decreased significantly. However, the most pronounced group differences were observed in sport-related physical activity, suggesting that structured exercise formats were particularly affected by the closure of rehabilitation centers and sports facilities. Reha participants experienced the largest reduction and reported higher perceived barriers such as lack of motivation and institutional restrictions. This finding is consistent with evidence indicating that patients with cardiovascular disease are especially vulnerable to interruptions in supervised rehabilitation programs [22,24].

Although activity levels declined, more than 85% of participants in all groups met WHO recommendations for moderate physical activity [8]. Nevertheless, healthy adults were significantly more likely to meet these recommendations compared to both patient groups. This contrasts with findings from other countries, where substantially lower proportions of individuals achieved recommended activity levels during lockdown [25,26]. Compensatory behaviors such as walking, cycling, or gardening may have contributed to maintaining moderate activity levels, as similarly reported in European populations during confinement periods [18,21].

### 4.3. Telemonitoring and Structured Support

An important observation concerns the KTx group, which remained enrolled in a telemonitoring-supported follow-up program during the lockdown period. Compared to Reha participants, KTx patients reported fewer perceived barriers and demonstrated a smaller decline in sporting activity.

In the pre–post subgroup analysis, sporting activity decreased in both KTx and HG participants during lockdown. However, overall activity increased in KTx patients due to higher levels of everyday and leisure activity, suggesting compensatory behavioral adaptation. As the pre-lockdown data were retrospectively obtained from routine assessments, causal inferences cannot be drawn. Nevertheless, the findings indicate that structured remote follow-up may be associated with smaller declines in activity levels during crisis situations.

This interpretation is supported by previous research suggesting that telemedicine-based programs and digital exercise interventions can help stabilize or promote physical activity under pandemic conditions [20,27]. Remote support formats have been proposed as viable strategies to counteract inactivity during quarantine periods [4,28,29]. The present results contribute to this body of evidence by demonstrating differential activity patterns between patients with and without ongoing structured support.

### 4.4. Correlation Between Physical Activity and Quality of Life

Correlation analyses revealed modest but statistically significant associations between total physical activity and physical QoL in the Reha (r = 0.22) and KTx (r = 0.19) groups. In healthy adults, PA was modestly associated with the mental sum score (r = 0.12). Although effect sizes were small, these findings are consistent with previous studies demonstrating that maintaining physical activity during lockdown is associated with better physical and psychological well-being [30,31,32].

Several studies conducted during the COVID-19 pandemic reported that individuals achieving recommended PA levels experienced fewer depressive symptoms and better overall mental health [30,32,33]. Physical activity has repeatedly been described as a protective factor against both physical deconditioning and psychological distress during quarantine situations [4,24,34]. Given the multifactorial determinants of QoL during a global health crisis—including social isolation, uncertainty, and economic stress—the modest magnitude of the observed correlations is plausible. Importantly, no significant pre–post changes in QoL were detected in the subgroup analysis, suggesting relative stability of perceived health status during the assessed period.

### 4.5. Clinical and Public Health Implications

The findings underscore the importance of maintaining accessible and adaptable exercise options for vulnerable patient groups during public health crises. Cardiac rehabilitation patients appear particularly dependent on supervised, structured exercise programs. Disruptions to such services may lead to disproportionate reductions in sporting activity and potentially unfavorable long-term cardiovascular consequences [24,35].

Telemonitoring-based follow-up programs, as implemented in the KTx group, may represent a promising strategy to support behavioral adaptation when in-person services are restricted [27,36]. Integrating flexible, hybrid rehabilitation models into routine care could enhance resilience in high-risk populations and reduce vulnerability to inactivity-related health risks during future crises.

### 4.6. Limitations

Several limitations should be considered. First, the primary study design was cross-sectional, limiting causal inference. Although a subgroup pre–post analysis was conducted, pre-lockdown data were retrospectively obtained from routine assessments and were not prospectively planned for this study. Second, physical activity was assessed using self-reported questionnaires, which are subject to recall bias and potential overestimation of moderate activity levels. Objective measurements were not feasible during lockdown conditions.

Third, recruitment was based on a convenience sampling strategy within a single academic center. Single-center studies may reflect regional or institutional characteristics and therefore have limited generalizability to broader populations [37]. Fourth, the healthy group differed in age and sample size compared to patient groups, which may have influenced between-group comparisons despite statistical testing.

Despite these limitations, the study provides valuable insight into behavioral adaptation during lockdown and highlights subgroups requiring targeted support during public health emergencies.

## 5. Conclusions

COVID-19 restrictions led to significant reductions in both daily and sporting physical activity across all groups, with the greatest decline observed among cardiac rehabilitation patients. Nevertheless, the majority of participants maintained recommended levels of moderate activity. Structured telemonitoring-supported follow-up programs were associated with smaller declines and compensatory behavioral adaptation.

Physical activity represents a key component of patient rehabilitation and is an essential element of holistic recovery. Even under lockdown conditions, the continuation of structured or home-based exercise should be considered a therapeutic priority for patients, as it directly supports the healing process and long-term functional outcomes. For healthy individuals, maintaining regular physical activity serves primarily as a preventive strategy, reducing the risk of lifestyle-related diseases and supporting overall physical and mental well-being.

Future public health strategies should therefore prioritize flexible, accessible, and digitally supported rehabilitation and exercise models to ensure continuity of physical activity in both vulnerable patient populations and the general population during crisis situations.

## Figures and Tables

**Table 1:** Group characteristics.

Parameters	Reha (*n* = 290)	KTx (*n* = 248)	HG (*n* = 985)	*p*-Value	Effect Size
Female/male	94/196 ^b^	89/159 ^c^	440/545 ^b,c^	<0.001	0.11
Age (years)	69.1 ± 10.7 ^a,b^	54.9 ± 13.4 ^a,c^	47.8 ± 16.0 ^b,c^	<0.001	0.24
Occupation					
No	185 (63.8%) ^a,b^	95 (38.5%) ^a,c^	133 (13.5%) ^b,c^	<0.001	0.34
Yes, sedentary	50 (17.2%) ^a,b^	69 (27.9%) ^a,c^	596 (60.6%) ^b,c^		
Yes, moderate activity	45 (15.5%) ^a,b^	77 (31.2%) ^a,c^	228 (23.2%) ^b,c^		
Yes, intense activity	10 (3.4%) ^a,b^	6 (2.4%) ^a,c^	26 (2.6%) ^b,c^		
Residential area					
Urban area	108 (37.2%) ^a,b^	41 (16.6%) ^a,c^	351 (35.6%) ^b,c^	<0.001	0.19
Suburban area	140 (48.3%) ^a,b^	73 (29.6%) ^a,c^	308 (31.3%) ^b,c^		
Rural area	42 (14.5%) ^a,b^	133 (53.8%) ^a,c^	326 (33.1%) ^b,c^		

^a^ *p* < 0.05 Reha vs. KTx, ^b^ *p* < 0.05 Reha vs. HG, ^c^ *p* < 0.05 KTx vs. HG.

**Table 2:** Results of the Freiburg questionnaire for physical activity during lockdown.

Parameters	Reha (*n* = 290)	KTx (*n* = 248)	HG (*n* = 984)	*p*-Value	Effect Size
Sum of activities (MET·h/week)	46.6 ± 40.4 ^b^	41.1 ± 35.2 ^c^	53.3 ± 41.6 ^b,c^	<0.001	0.14
Every day PA (MET·h/week)	19.6 ± 21.9	19.8 ± 21.8	21.2 ± 21.6	0.411	-
Leisure-time PA (MET·h/week)	21.4 ± 23.3 ^a^	16.4 ± 22.0 ^a,c^	21.2 ± 23.8 ^c^	0.008	0.01
Sport-related physical activity (MET·h/week)	5.6 ± 14.7 ^b^	5.0 ± 10.2 ^c^	10.9 ± 24.4 ^b,c^	<0.001	0.02
Classification					
Below PA recommendations	46 (15.9%) ^b^	58 (23.4%) ^c^	78 (7.9%) ^b,c^	<0.001	0.14
Minimum PA recommendations achieved	70 (24.1%) ^b^	53 (21.4%) ^c^	176 (17.9%) ^b,c^		
Meeting PA recommendations	174 (60.0%) ^b^	137 (55.2%) ^c^	730 (74.2%) ^b,c^		

^a^ *p* < 0.05 Reha vs. KTx, ^b^ *p* < 0.05 Reha vs. HG, ^c^ *p* < 0.05 KTx vs. HG.

**Table 3:** Time spend in moderate and intensive activity during lockdown.

Parameters	Reha (*n* = 290)	KTx (*n* = 248)	HG (*n* = 985)	*p*-Value	Effect Size
Moderate PA (min/week)	693 ± 610	635 ± 538	673 ± 522	0.442	-
Moderate sport-related PA (min/week)	40 ± 97	38 ± 95	35 ± 72	0.619	-
Intensive sport-related PA (min/week)	23 ± 95 ^b^	18 ± 57 ^c^	66 ± 187 ^b,c^	ok	0.02

^b^ *p* < 0.05 Reha vs. HG, ^c^ *p* < 0.05 KTx vs. HG.

**Table 4:** Health-related quality of life during lockdown (in parenthesis German normative data as 95% confidence interval [17].

Parameters	Reha (*n* = 290)	KTx (*n* = 248)	HG (*n* = 985)	*p*-Value	Effect Size
Physical sum score	44.0 ± 10.1 ^a^ (46.3–47.6)	44.4 ± 10.5 ^b^ (49.9–51.0)	55.5 ± 4.8 ^a,b^ (51.9–52.9)	<0.001	0.36
Mental sum score	48.8 ± 9.0 (50.5–51.9)	49.2 ± 10.4 (48.4–49.8)	49.7 ± 8.0 (48.1–49.5)	0.263	-

^a^ *p* < 0.05 Reha vs. HG, ^b^ *p* < 0.05 KTx vs. HG.

**Table 5:** Physical activity and health-related quality of life of KTx and HG within a year prior to the survey and during lockdown.

Parameters	KTX (*n* = 179)		HG (*n* = 62)		*p*-Value	Effect Size
	Pre	Post	Pre	Post		
Total weekly PA (MET·h/week)	30.8 ± 30.1	41.9 ± 37.0 *	49.9 ± 37.0	44.8 ± 32.9	0.004	0.03
Habitual daily PA (MET·h/week)	12.1 ± 13.8	21.0 ± 22.5 *	15.1 ± 17.6	12.7 ± 14.8	<0.001	0.05
Leisure-time PA (MET·h/week)	9.6 ± 11.0	16.1 ± 23.0 *	10.9 ± 15.8	14.3 ± 14.1	0.343	-
Sport-related PA (MET·h/week)	9.2 ± 20.4	4.8 ± 9.6 *	24.0 ± 18.2	17.8 ± 21.0 *	0.542	-
Quality of life						
Physical sum score	43.7 ± 10.6	45.2 ± 10.2	55.1 ± 8.9	56.8 ± 4.3 *	0.892	-
Mental sum score	50.6 ± 10.3	48.7 ± 10.8	51.3 ± 8.3	49.7 ± 8.7	0.780	-

Pre: within a year prior to the survey; post: during lockdown. * *p* < 0.05 pre vs. post.

## Data Availability

The original contributions presented in this study are included in the article. Further inquiries can be directed to the corresponding author.

## Supplementary Materials

The following supporting information can be downloaded at: https://www.mdpi.com/article/10.3390/ijerph23030323/s1, Supplementary File S1: English language version of the questions developed for this study (original in German).
