# Supplementary material for: Influence of Restrictions During COVID-19 Pandemic on Physical Activity and Quality of Life in Cardiovascular, Kidney Transplant and Healthy Adults
Source: Int J Environ Res Public Health. 2026 Mar 5;23(3):323. doi: 10.3390/ijerph23030323 (PMC13026377; doi:10.3390/ijerph23030323)
Supplement: Supplementary file 1 [file ijerph-23-00323-s001.zip › ijerph-4124734-supplementary.pdf]

Supplementary file 1:

English language version of the questions developed for this study (original in German):

Questionnaire on Physical Activity, Restrictions, and Burdens Due to the Corona (SARS-CoV-2) Pandemic

Dear Sir or Madam,

The following questionnaire will ask you some questions about your activity behavior and the restrictions and burdens caused by the Corona (SARS-CoV-2) pandemic. Please note that some questions refer to the last week and others to the last month. The first questions regarding daily activities are divided into commuting to work and leisure activities. The questions about sports (activities for which you wore sports clothes) come after the daily activities. Completing the questionnaire takes approximately 15 minutes.

Are you employed (including housewife) or in education?

☐ No

☐ Yes

- ☐ Sedentary activity (e.g. office work, student…)
- ☐ Moderate activity (e.g. craftsmen, housewife…)
- ☐ Intense activity (e.g. postmen, construction worker…)

How would you describe your living environment?

☐ I live in an urban area

☐ I live in a residential area on the outskirts of the city

☐ I live in a rural area

How has your daily activity changed due to the current corona restrictions? (Everyday and leisure activities)

Everyday activities should be considered here. You **did not put on sports clothes** for these activities.

My <u>daily activity</u> before the restrictions:

Looking back, how would you rate your activity level before the Corona pandemic? (0= no activity, 10= very high activity)

☐	☐	☐	☐	☐	☐	☐	☐	☐	☐	☐
0	1	2	3	4	5	6	7	8	9	10

How would you currently rate your activity level?

(0= no activity, 10= very high activity)

☐	☐	☐	☐	☐	☐	☐	☐	☐	☐	☐
0	1	2	3	4	5	6	7	8	9	10

If your activity level has changed during the Corona pandemic period, please indicate below how and in which areas changes have occurred.

Reduction in daily activity due to:

Please answer this question if you are exercising **less** in your daily life **than before**!

☐ Less time

☐ Lack of motivation

☐ Restrictions

☐ Other burdens

Increase in daily activity due to:

Please answer this question if you are exercising **more** in your daily life **than before**!

☐ More time

☐ More gardening

☐ More walks

☐ Switch to a bike

How has your sporting activity changed due to the current Corona restrictions? (Endurance training, strength training, other sports and rehabilitation sports)

Sporting activities should be considered here. For these activities you should have put on **sports clothes**.

My sporting activity before the restrictions:

Looking back, how would you rate your activity level before the Corona pandemic? (0= no activity, 10= very high activity)

☐	☐	☐	☐	☐	☐	☐	☐	☐	☐	☐
0	1	2	3	4	5	6	7	8	9	10

My sporting activity at the end of lockdown:

How do you self-assess your activity level? (0= no activity, 10= very high activity)

☐	☐	☐	☐	☐	☐	☐	☐	☐	☐	☐
0	1	2	3	4	5	6	7	8	9	10

If your activity level has changed during the Corona pandemic period, please indicate below how and in which areas changes have occurred.

Reduction in sporting activity due to:

Please answer this question if you are **less active in sports**!

☐ Less time

☐ Lack of motivation

☐ Restrictions

☐ Gym/ sports club closed

Increase in sporting activity by:

Please answer this question if you are **more active in sports**!

☐ More time

☐ Switch to a bike

☐ Home workout

☐ Use of digital sports offers (video workout)
